# Reply to Comment on: “The impact of psychiatric utilisation prior to cancer diagnosis on survival of solid organ malignancies’’

**DOI:** 10.1038/s41416-019-0494-6

**Published:** 2019-06-10

**Authors:** Zachary Klaassen, Christopher J. D. Wallis, Paul Kurdyak, Girish S. Kulkarni

**Affiliations:** 1 Division of Urology, Medical College of Georgia – Augusta University, Augusta, GA USA; 2 Georgia Cancer Center, Augusta, GA USA; 3 Department of Surgery, Division of Urology, University of Toronto, University Health Network, Princess Margaret Cancer Centre, Toronto, ON Canada; 4 Institute for Health Policy, Management and Evaluation, University of Toronto, Toronto, ON Canada; 5 Institute for Clinical Evaluative Sciences, Toronto, ON Canada; 6 Institute for Mental Health Policy Research, Centre for Addiction and Mental Health, Toronto, ON Canada

**Keywords:** Cancer, Surgical oncology

We thank Professor Braillon for his comment on our article *The impact of psychiatric utilisation prior to cancer diagnosis on survival of solid organ malignancies.*^1^ Prof. Braillon noted that (i) vulnerable people (i.e. those with psychiatric comorbidities) are more prone to be victims than aggressors, whereby patients with psychiatric utilisation are not actively involved in an unhealthy activity, as suggested by the term “engage”; and (ii) little progress for implementing smoking cessation for patients with serious mental illness is not restricted to psychiatry. These points deserve greater context with regard to the goals and findings in our study.

Indeed, we note in our discussion that patients with psychiatric comorbidities may be marginalised (i.e. vulnerable victims) with regard to their care, as Prof. Braillon also highlighted. However, regardless of verbiage or semantics, these patients are more likely to demonstrate behaviours, such as smoking or alcohol use, which may be detrimental to their physical health. For instance, in the United States, people with mental illness consume a disproportionately higher quantity of tobacco compared with the general population.^2^ Further, between 2004 and 2011, the decline in smoking among individuals with mental illness was significantly less than among those without mental illness.^3^ Among 9142 people with schizophrenia, schizoaffective disorder or bipolar disorder with psychotic features matched to 10,195 controls, those with severe mental illness were four times (OR 4.0, 95% CI 3.6–4.4) more likely to be heavy alcohol users (≥4 drinks per day).^4^ We highlight these statistics not to cast blame on these individuals or to further marginalise them, but rather to bring to light these comorbidities among oncology patients with psychiatric comorbidities—a component that may be contributing to our findings of worse cancer and all-cause mortality among patients with psychiatric utilisation.

Second, we agree that there is poor implementation of smoking cessation programmes among not only patients with serious mental illness, but also oncology patients. Among 27,157 individuals interviewed for the 2010 National Health Interview Survey, only 51.7% individuals diagnosed with cancer who were active smokers reported being counselled to quit smoking by a health professional within the previous 12 months.^5^ Certainly, this is of concern; however so is the fact that in this study, cancer survivors were no more likely to quit smoking than individuals in the general population, regardless of the smoking cessation method used.^5^ Furthermore, in a systematic review of smoking cessation studies (using interventions such as counselling, nicotine replacement therapy, bupropion and varenicline) among oncology patients, intervention had an odds ratio for success of 1.54 (95% CI 0.91–2.64) for patients in a short follow-up group, and a similar statistically non-significant efficacy among patients in a longer follow-up group (OR 1.31, 95% CI 0.93–1.84).^6^ These studies highlight that we are not counselling oncology patients and even when we do, the methods are ineffective. There is no question that this is an important area that requires future attention to develop efficacious and sustainable long-term cessation interventions.

As noted in our discussion,^1^ this is an epidemiological study suggesting an association between psychiatric utilisation pre-diagnosis and worse cancer and all-cause mortality outcomes—this is not a “cause and effect” study. However, our results suggest that there is an effect gradient for worse cancer-specific mortality across the intensity of pre-diagnosis psychiatric utilisation (vs. no psychiatric utilisation): 5% increased risk for outpatient psychiatric utilisation (HR 1.05, 95% CI 1.04–1.06), 36% for emergency department psychiatric utilisation (HR 1.36, 95% CI 1.30–1.42) and 73% for inpatient psychiatric utilisation (HR 1.73, 95% CI 1.63–1.84). Whether these patients (i) have biological changes that portend a worse cancer diagnosis, (ii) are less likely to adhere to follow-up schedules and more likely to engage in behaviours that are detrimental to overall health, (iii) may not be receiving adequate screening or (iv) are marginalised by physicians and receive substandard care that deviates from established guidelines—it is our responsibility as a healthcare community to identify these disparities and provide meaningful interventions in order for these patients to achieve survival outcomes comparable with those without psychiatric comorbidities.

## Data Availability

Not applicable.
